# Pain anticipation is a new behavioural sign of minimally conscious state

**DOI:** 10.1093/braincomms/fcae311

**Published:** 2024-09-16

**Authors:** Aude Sangare, Esteban Munoz-Musat, Amina Ben Salah, Melanie Valente, Clemence Marois, Sophie Demeret, Jacobo Diego Sitt, Benjamin Rohaut, Lionel Naccache

**Affiliations:** Paris Brain Institute-ICM, Inserm U1127, CNRS UMR 7225, PICNIC Lab, Sorbonne Universite, Paris 75013, France; Département de Neurophysiologie, Sorbonne Université, Assistance Publique—Hôpitaux de Paris, Groupe Hospitalier Pitié-Salpêtrière Charles Foix, Paris 75013, France; Paris Brain Institute-ICM, Inserm U1127, CNRS UMR 7225, PICNIC Lab, Sorbonne Universite, Paris 75013, France; Paris Brain Institute-ICM, Inserm U1127, CNRS UMR 7225, PICNIC Lab, Sorbonne Universite, Paris 75013, France; Paris Brain Institute-ICM, Inserm U1127, CNRS UMR 7225, PICNIC Lab, Sorbonne Universite, Paris 75013, France; Département de Neurophysiologie, Sorbonne Université, Assistance Publique—Hôpitaux de Paris, Groupe Hospitalier Pitié-Salpêtrière Charles Foix, Paris 75013, France; Département de Neurologie, Sorbonne Université, Assistance Publique—Hôpitaux de Paris, Groupe Hospitalier Pitié-Salpêtrière Charles Foix, médecine intensive et réanimation Paris, Paris 75013, France; Département de Neurologie, Sorbonne Université, Assistance Publique—Hôpitaux de Paris, Groupe Hospitalier Pitié-Salpêtrière Charles Foix, médecine intensive et réanimation Paris, Paris 75013, France; Paris Brain Institute-ICM, Inserm U1127, CNRS UMR 7225, PICNIC Lab, Sorbonne Universite, Paris 75013, France; Paris Brain Institute-ICM, Inserm U1127, CNRS UMR 7225, PICNIC Lab, Sorbonne Universite, Paris 75013, France; Département de Neurologie, Sorbonne Université, Assistance Publique—Hôpitaux de Paris, Groupe Hospitalier Pitié-Salpêtrière Charles Foix, médecine intensive et réanimation Paris, Paris 75013, France; Paris Brain Institute-ICM, Inserm U1127, CNRS UMR 7225, PICNIC Lab, Sorbonne Universite, Paris 75013, France; Département de Neurophysiologie, Sorbonne Université, Assistance Publique—Hôpitaux de Paris, Groupe Hospitalier Pitié-Salpêtrière Charles Foix, Paris 75013, France

**Keywords:** disorder of consciousness, pain anticipation, intensive care, EEG, trace conditioning

## Abstract

Probing cognition and consciousness in the absence of functional communication remains an extremely challenging task. In this perspective, we imagined a basic clinical procedure to explore pain anticipation at bedside. In a series of 61 patients with a disorder of consciousness, we tested the existence of a nociceptive anticipation response by pairing a somaesthetic stimulation with a noxious stimulation. We then explored how nociceptive anticipation response correlated with (i) clinical status inferred from Coma Recovery Scale-Revised scoring, (ii) with an EEG signature of stimulus anticipation—the contingent negative variation—and (iii) how nociceptive anticipation response could predict consciousness outcome at 6 months. Proportion of nociceptive anticipation response differed significantly according to the state of consciousness: nociceptive anticipation response was present in 5 of 5 emerging from minimally conscious state patients (100%), in 10 of 11 minimally conscious state plus patients (91%), but only in 8 of 17 minimally conscious state minus patients (47%), and only in 1 of 24 vegetative state/unresponsive wakefulness syndrome patients (4%) (*χ*^2^  *P* < 0.0001). Nociceptive anticipation response correlated with the presence of a contingent negative variation, suggesting that patients with nociceptive anticipation response were more prone to actively expect and anticipate auditory stimuli (Fisher’s exact test *P* = 0.05). However, nociceptive anticipation response presence did not predict consciousness recovery. Nociceptive anticipation response appears as a new additional behavioural sign that can be used to differentiate minimally conscious state from vegetative state/unresponsive wakefulness syndrome patients. As most behavioural signs of minimally conscious state, the nociceptive anticipation response seems to reveal the existence of a cortically mediated state that does not necessarily reflect residual conscious processing.

## Teaser

Sangare *et al*. studied 61 patients with a disorder of consciousness. They tested a basic clinical procedure to explore the preservation of nociceptive anticipation responses at bedside by pairing neutral somaesthetic stimulation with noxious stimulation. The presence of nociceptive anticipation responses differed significantly according to the state of consciousness.

## Graphical Abstract

**Graphical Abstract:**
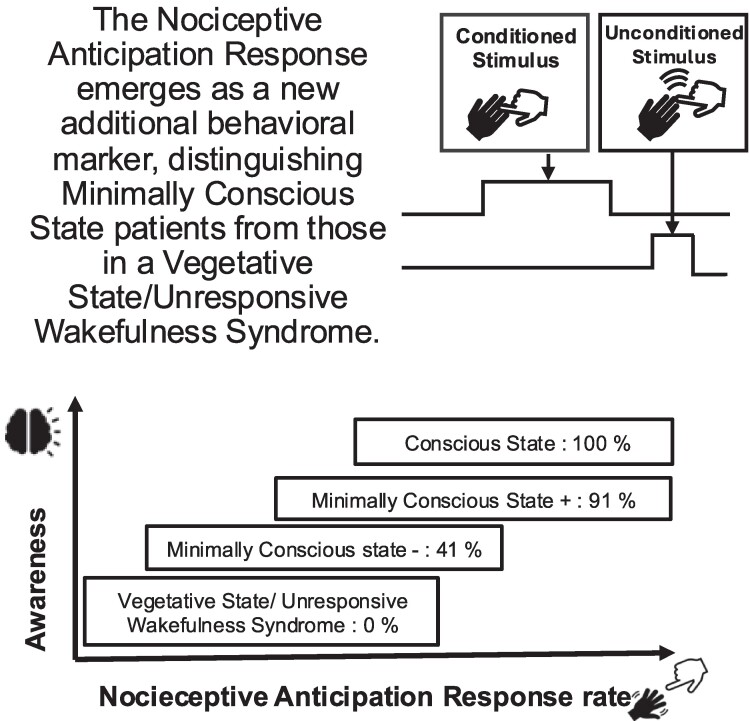

## Introduction

Probing cognition and consciousness in the absence of functional communication remains an extremely challenging task. Such a challenge is faced every day by clinicians and caregivers interacting with awake but non-communicating patients regrouped under the generic label of disorders of consciousness (DoC). A careful and repeated behavioural examination allows the distinction between the vegetative state, also coined the unresponsive wakefulness syndrome (VS/UWS), and the minimally conscious state (MCS) in which non-reflexive cognitively mediated behaviours demonstrate a richer state associated with richer cortical processing.^1^ MCS was recently subcategorized on the basis of the absence/presence of signs of language function: MCS+ patients describe high-level behavioural responses (i.e. command-following, intentional although non-functional communication) and MCS− patients only show contextualized motor and emotional behaviours (i.e. visual pursuit, localization of noxious stimulation).^2^

Still, many studies converge to show that sole behavioural examination is mandatory but insufficient: up to 15–20% of patients appear to be in a VS/UWS, whereas their brain activity is suggestive of a MCS or even of a conscious state.^3^

In this perspective, enrichment of the behavioural repertoire that can be tested at bedside with simple clinical methods appears as a priority goal (for instance, see Arzi *et al*.,^4^ Chatelle *et al*.,^5^ Hermann *et al*.,^6^ and Schnakers *et al*.^7^).

In the present work, we aimed at conceiving a new simple bedside behavioural test that could probe a new type of cognitively mediated behaviour inspired by the literature of nociception anticipation and conditioning: we imagined a basic clinical procedure to explore pain anticipation at bedside. A key distinction has been discovered between delay conditioning on the one hand and trace conditioning on the other hand.^8,9^ In a delay conditioning paradigm, a neutral conditioned stimulus (CS) is delivered prior to the onset of an aversive or noxious unconditioned stimulus (US), but both stimuli overlap in time. In contrast, trace conditioning paradigms are defined by the insertion of a temporal gap > 1 s between CS and US.

Trace conditioning can be disrupted by a distractor provided during the gap separating the CS and the US.^10^ There is no trace conditioning without report of the contingency between CS and US raising the question of whether trace conditioning may reveal the presence of conscious processing^11-13^ as necessary states to bridge the gap between the CS and the US.

**Figure 1:**
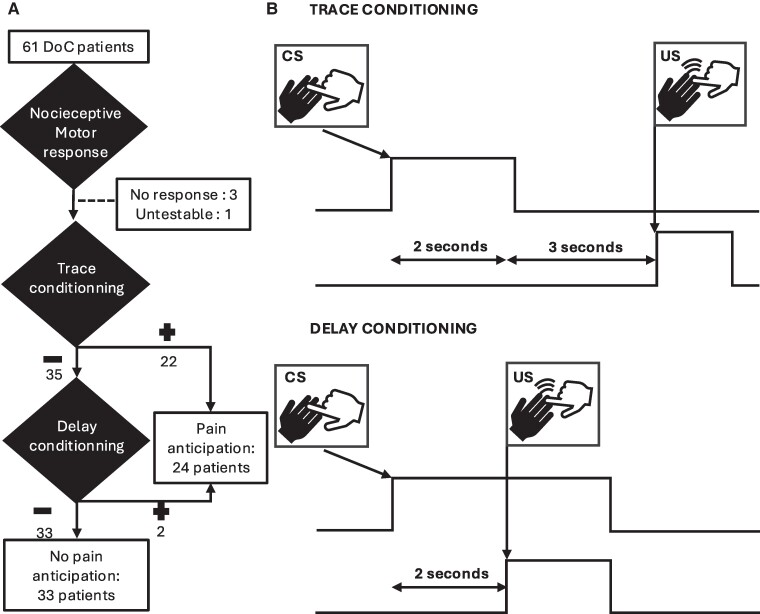
**Flowchart (A) and stimulus design (B): in the trace-conditioning paradigm, we gently touched the middle finger of the patient for about 2 s (neutral CS), and then, after a SOA of about 3 s, we applied a noxious pressure on the nail bed of the middle finger of the same hand (aversive or noxious US).** In the ‘delay-like’ conditioning paradigm, we delivered the same stimuli but without delay between the end of the tactile stimulation and the noxious stimulation.

Inspired by this literature, we designed the pain anticipation test and applied it to a series of 61 DoC patients: we first checked the preservation of behavioural reactivity to unconditioned noxious stimuli. Then, we used a hierarchical procedure by pairing a tactile stimulation (i.e. that would play here the role of a CS) with a noxious stimulation (i.e. that would play here the role of an US) delivered to the same body location: we tested the existence of a nociceptive trace-like anticipation response (NTAR; see Fig. 1 and *Materials and methods*). In case of absence of NTAR, patients were finally tested for basic nociceptive delay-like anticipation response (NDAR), by overlapping in time the initial CS tactile stimulation with the upcoming US noxious stimulation.

We then explored how nociceptive anticipation responses (NARs) correlated with (i) clinical status inferred from Coma Recovery Scale-Revised (CRS-r) scoring,^14^ (ii) with an EEG signature of stimulus anticipation: the contingent negative variation (CNV),^15^ elicited by the auditory local global paradigm,^16^ and (iii) how NAR could predict conscious outcome at 6 months.

Our main prediction was that NTAR should require a functional GNW architecture and should therefore be specific to patients identified as being conscious or MCS+, both from behavioural and EEG data.

## Materials and methods

### Ethics statement

This research was approved by the local ethics committee ‘Comité de Protection des Personnes Ile de France 1 (Paris, France)’ under the code ‘Recherche en soins courants’ (M-NEURODOC protocol). Patient’s family gave their informed consent for the participation of their relative, and all investigations conformed to the Declaration of Helsinki and the French regulations.

### Participants

All participants referred for an evaluation of consciousness to the neurology intensive care unit of Pitié-Salpêtrière University Hospital (APHP and Sorbonne University) from January 2020 and July 2022 were screened for participation in the study. Inclusion criteria were (i) age between 18 and 80 years (ii) with a disorder of consciousness as assessed by the CRS-r (VS, MCS, or exit-MCS). Exclusion criteria were (i) the absence of motor response to pain and (ii) major oppositional behaviour.

In addition to the new behavioural test stemming from conditioning paradigms, patients’ evaluation included neurological examination, Nociception Coma Scale-revised (NCS-r)^17^ and CRS-r^14^ scoring carried out at least three times by trained clinicians. The presence/absence of sedative drugs or neurotropic drugs was collected from medical records. Note that the limited size of our sample prevented us to analyse the impact of comorbidities on NAR.

### Conditioning response procedure

We used the following hierarchical procedure inspired by conditioning literature mentioned above. Note that this pain anticipation protocol (PAP) inspired by trace versus delay conditioning tests was conceived to be applicable in routine bedside testing, but that it does not strictly correspond to a classical conditioning paradigm.

For ethical reasons, patients were warned that their responses to unpleasant stimuli were going to be tested. First, each patient was tested for the presence of a clear behavioural response (such as localization of noxious stimulus response, flexion withdrawal, or abnormal posturing response) to noxious stimulation of the nail bed of one of their middle fingers. In the absence of any such nociceptive response that may correspond to sensory deficits, the procedure was stopped, and the patient was excluded from the conditioning tests (see Fig. 1).

Second, when a patient showed a behavioural response to nociceptive behaviour, we then looked for a ‘trace-like’ conditioning response to the hand with the richest motor response in case of asymmetry: we gently touched the middle finger of the patient for about 2 s, and then after a stimulus onset asynchrony (SOA) of 3 s, we applied a noxious pressure on the nail bed of the middle finger of the same hand. The fingernail pressure was stopped as soon as a behavioural response was observed. At the end of the unconditioned behavioural response, we waited for 3 s and then repeated the same sequence while looking for a behavioural response during the CS condition. As soon as a CS response was present, this test was stopped. In the absence of behavioural response during the CS, we repeated the US. In the presence of a CS response (i.e. on the first, second, or third trial), the patient was labelled as positive for the NTAR conditioning test, whereas he/she was labelled as negative for this test in the absence of CS response after three successive trials.

Third, in the absence of ‘trace-like’ conditioning, we exposed the patient to a ‘delay-like’ conditioning test, by delivering the same stimuli (CS then US) but without delay between the end of the tactile stimulation and the noxious stimulation. We repeated the sequence up to three times: as soon as a CS response was observed, the patient was labelled as positive for this NDAR conditioning test, or negative if he/she did not show any such CS response after three successive trials.

Finally, a patient with any of these two responses (NTAR or NDAR) was labelled as showing a NAR according to this new PAP.

### Other behavioural measures

In addition to NAR, we also systematically collected (i) the habituation of auditory startle response (hASR),^6^ (ii) the CRS-r^14^ and (iii) the (NCS-r)^5^ according to standardized procedures.

For hASR, we followed instructions reported in Hermann *et al*.:^6^ ASR was assessed presenting 10 times a loud noise by clapping your hands directly above the patient’s head and out of view. The reflex was considered inextinguishable, if there is an eyelid blink following each and every clap. Otherwise, the reflex was considered extinguishable.

The CRS-r was used to define state of consciousness on the day of the test. Inclusion criteria included the presence of a standard pain response. Six-month outcome was collected through semi-structured phone interview of the treating physician and/or of the family performing a CRS-r–like assessment and reporting their observations. In accordance with the CRS-r criteria, consciousness recovery was defined by accurate functional communication or by the functional use of tools.

### Event-related potentials

High-density scalp EEG was recorded at the bedside, on the same day as the clinical assessment (CRS-r and conditioning paradigm) using the ‘local global’ auditory oddball paradigm.^16^ We specifically aimed at correlating our NAR behavioural results with the contingent negative variation (CNV) event-related potential (ERP) component previously characterized in this task. CNV corresponds to a slow anterior midline negative drift beginning from the onset of the first sound to the onset of the fifth sound, indexing cognitive expectancy of the fifth sound.^15,18^ We also computed the presence of an ERP local or global effect as described in our previous studies .^16,19^

Recordings were made at a 250 Hz sampling frequency using a-256 electrode HydroCel Geodesic Sensor Net (Electrical Geodesics) referenced to the vertex with impedances set below 10 kΩ prior to acquisition. Pre-processing and EEG analysis methods were similar to Sitt *et al*.^20^ In short, epochs were baseline-corrected over the 200 ms preceding the onset of the first sound, and we computed the slope of the ordinary least-squares regression with patients’ average voltage. At the individual level, a linear regression was calculated for each individual trial on the vertex centred region of interest during the 0–600 ms time window of interest. Then, the distribution of these individual trials’ slopes was compared to zero with a one-sample Student *t*-test. To be accounted for, the slope had to be statically negative and at the scalp level respect an antero-posterior topography.

### Statistical analysis

Population characteristics were described using the median interquartile range (IQR). Group differences were tested by two-sample Mann–Whitney *U*-test and χ^2^ test. Diagnostic performance of the conditioning paradigm was assessed using standard classification metrics [sensitivity, specificity, predictive values, likelihood ratios, accuracy, and area under the receiver operating characteristics (ROC) curve (AUC)] with their 95% confidence intervals (95% CIs).

## Results

Between January 2020 and July 2022, 61 DoC patients were screened. Among them, three patients were excluded because they lacked any motor response to noxious stimuli, and one additional conscious patient in an emergence from MCS (EMCS) was excluded because of major oppositional behaviour leading to systematic withdrawal to any stimulation (i.e. including withdrawal to non-noxious light tactile stimulation). In total, 57 patients were included and tested with the pain anticipation paradigm (24 in a VS/UWS; 17 in a MCS−; 11 in a MCS+; and 5 in an EMCS).^21^ Patients’ main characteristics are shown in Table 1, and further details are presented in Supplementary Table 1.

**Table 1:** Population characteristics

	All (*n* = 57)	Nociceptive anticipation (*n* = 24)	Absence of nociceptive anticipation (*n* = 33)	*P*
Demographic characteristics				
▪ Age, years	51 [25–59]	54 [34–60]	49 [37–58]	0.5
▪ Sex ratio, male-to-female	24/26	14/10	20/13	0.9
Time since injury				
▪ Days	52 (28–108)	88 (41–132)	42 (26–76)	0.1
▪ Chronic	22 (39%)	12 (50%)	10 (30%)	0.3
▪ Acute	33 (61%)	12 (50%)	23 (70%)	
Aetiology				0.6
▪ Anoxia	21 (37%)	7 (29%)	14 (42%)	
▪ Traumatic	10 (18%)	7 (29%)	3 (9%)	
▪ Vascular	10 (18%)	2 (8%)	8 (24%)	
▪ Infectious	6 (11%)	4 (17%)	2 (6%)	
▪ Dysimmune encephalitis	3 (5%)	1 (4%)	2 (6%)	
▪ Hypoglycaemia				
▪ Epilepsia	3 (5%)	0 (0%)	3 (9%)	
▪ Other	2 (4%)	2 (8%)	0 (0%)	
	2 (4%)	1 (4%)	1 (3%)	
State of consciousness				<0.0001
▪ VS/UWS	24 (42%)	1 (4%)	23 (70%)	
▪ MCS–	17 (30%)	8 (33%)	9 (27%)	
▪ MCS+	11 (19%)	10 (42%)	1 (3%)	
▪ EMCS	5 (9%)	5 (21%)	0	
CRS-r				
▪ Total score	9 (6–12)	14 (11–17)	6 (5–8)	<0.0001
▪ Audio sub-score	1.5 (1–3)	3 (2–3)	1 (1–1)	<0.0001
▪ Visual sub-score	1.5 (1–3)	3 (3–4.5)	0 (0–1)	<0.0001
▪ Motor sub-score	2 (2–5)	4 (2–5)	2 (2–2)	0.0015
▪ Verbal sub-score	1 (1–1)	1 (1–2)	1 (1–1)	0.0008
▪ Communication sub-score	0 (0–0)	0 (0–1)	0 (0–0)	<0.0001
▪ Arousal sub-score	2 (1–2)	2 (2–3)	2 (1–2)	<0.0001
NCS-r				
▪ Total score	4 (3–5)	5 (5–7)	3 (2–4)	<0.0001
▪ Motor response	2 (2–3)	3 (2–3)	2 (1–2)	<0.0001
▪ Verbal response	0 (0–1)	1 (0–2)	0 (0–0)	<0.0001
▪ Facial response	2 (2–3)	2 (2–2)	1 (0–2)	<0.0001
Habitation of auditory startle				0.009
▪ Extinguishable	36 (63%)	22 (91%)	14 (42%)	
▪ Inextinguishable	17 (30%)	2 (9%)	15 (45%)	
▪ Absence	4 (7%)	0	4 (12%)	

Quantitative data are expressed as median (IQR) and compared with Mann–Whitney *U*-tests. Categorical data are expressed as *n* (%) and compared with *χ*^2^ tests.

A NAR resulting in a withdrawal of the hand during the neutral stimulus (i.e. NDAR or NTAR) was observed in 24 patients (42%), while no pain anticipation could be observed in the remaining 33 patients (58%; see flowchart in Fig. 1). Note that we classified within the ‘no pain anticipation’ group the two patients in whom noxious stimulation elicited a grimace without hand withdrawal. Demographic characteristics were comparable between the two groups (see Table 1 and Supplementary Table 1).

Interestingly, validity of our simplified bedside procedure inspired by and stemming from conditioning literature was confirmed by the absence of any hierarchical mismatch: no patient was found positive for the cognitively more-demanding NTAR behaviour, while being negative for the less-demanding NDAR behaviour.

### Pain anticipation behaviour is a new reliable MCS item

In clear agreement with our main prediction, NTAR was observed exclusively in conscious (i.e. EMCS) or in MCS patients: none of the 24 patients in a VS/UWS showed a NTAR (Fisher’s exact test *P* < 0.0001). Moreover, 100% of univocally conscious patients (i.e. EMCS) showed a NTAR, and NTAR was significantly more frequent in MCS+ (10/11 patients) than in MCS− patients (7/17 patients; Fisher’s exact test *P* = 0.016).

**Figure 2:**
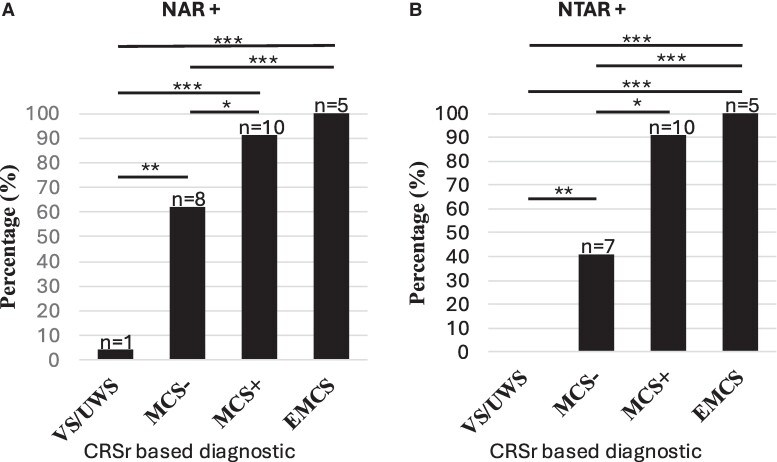
**(A) NAR+ and (B) NTAR+.** EMCS, emerging from minimally conscious state; MCS+, minimally conscious state plus; VS/UWS, vegetative state/unresponsive wakefulness syndrome. *P< 0.05, **P < 0.01, ***P < 0.001, Fischer’s exact test.

Interestingly, only two patients lacking a NTAR showed a NDAR (one VS/UWS patient and one MCS− patient; see Supplementary Table 1). We therefore gathered NTAR and NDAR in the generic category of NAR (see Fig. 2) that seems mostly driven by high-level trace-like anticipation behaviour in the present extensive group of DoC patients.

NAR appeared as a strong diagnostic predictor of MCS. NAR was present in 5 of 5 EMCS patients (100%), in 10 of 11 (91%) MCS+ patients, but only in 8 of 17 MCS− patients (62%), and only in 1 of 24 VS/UWS patients (4%) (*χ*^2^ = 35; *P* < 0.0001; see Fig. 2). Likewise, patients with NAR had higher scores in every CRS-r and NCS-r subscales.

By dichotomizing DoC patients between MCS and VS/UWS patients, proportion of NAR differed significantly between MCS and VS patients: NAR was present in 18 of the 28 (64%) MCS patients and in only 1 of the 24 (4%) VS patients (Fisher’s exact test *P* < 0.0001); AUC for the discrimination of MCS from VS/UWS was 0.79%, with 95% CI 0.66–0.89; sensitivity = 64% (44–81); specificity = 96% (80–99); positive predictive value = 95% (72–99); negative predictive value = 71% (59–80); positive likelihood ratio = 16 (2–112); and negative likelihood ratio = 0.37 (0.22–0.62)).

Presence of NAR performed close to other CRS-r discriminative items (Table 2). This is remarkable, because, contrarily to CRS-r MCS items that are by definition incorporated to this scale, this new behavioural sign is not circularly used to defined MCS and VS/UWS. In the same perspective, NAR performed also close to the presence of extinguishable ASR^6^ and close to the presence of a NCS-r score ≥ 4 [according to the threshold proposed by Chatelle *et al*.^5^ (Table 2)]. Interestingly NCS-r scores were strongly correlated to states of consciousness with higher values in MCS patients than in VS/UWS patients [VS/UWS median NCS-r = 3, quartile1 (Q1) = 1, Q3 = 3; MCS median NCS-r = 5, Q1 = 4, Q3 = 8]. Note that among the different NCS-r thresholds proposed, the highest AUC to discriminate MCS from VS/UWS patients was obtained with a cut-off score ≥ 4 [AUC for the discrimination of MCS from VS/UWS was 0.87%, with 95% CI 0.74–0.94; sensitivity = 93% (77–99); specificity = 79% (59–93); positive predictive value = 84% (70–92); negative predictive value = 90% (71–97)]. However, as previously described,^22,23^ a cut-off score of 2 was very sensitive to diagnose MCS state and a cut-off score of 5 was very specific (NCS-r score ≥ 2 AUC 0.67%, with 95% CI 0.53–0.80; sensitivity = 100% (88–100); specificity = 29% (13–51); positive predictive value = 62% (56–68); negative predictive value = 100% (59–100); NCS-r score ≥ 5 AUC 0.73%, with 95% CI 0.59–0.84; sensitivity = 54% (34–72); specificity = 96% (79–99); positive predictive value = 94% (68–99); negative predictive value = 64% (64–73)].

**Table 2:** Performances of NAR and NTAR versus other clinical signs to discriminate MCS from VS/UWS patients

	Pr (%)	AUC (95% CI)	Sen % (95% CI)	Sp % (95% CI)	PPV % (95% CI)	NPV% (95% CI)	PLR (95% CI)	NLR (95% CI)
NAR	37	79 (66–89)	64 (44–81)	96 (80–99)	95 (72–99)	71 (59–80)	16 (2–112)	0.37 (0.2–0.6)
NTAR	34	79 (65–89)	61 (41–79)	100 (86–100)	100 (80–100)	69 (58–78)		0.39 (0.3–0.6)
Ext ASR	61	73 (59–84)	82 (63–94)	63 (41–81)	72 (60–82)	75 (56–86)	2.2 (1.2–3.4)	0.29 (0.1–0.7)
NCS-r (≥4)	59	87 (74–94)	93 (77–99)	79 (58–93)	84 (70–92)	90 (71–97)	5 (2–10)	0.09 (0.02–04)
Auditory ≥ 3 reproducible	21	67 (53–80)	39 (22–59)			59 (51–66)		0.61 (0.5–0.8)
Visual ≥ 2 fixation	46	92 (81–98)	86 (67–96)			86 (71–93)		0.14 (0.1–0.4)
Motor ≥ 3 localization	31	77 (63–87)	57 (37–67)			67 (57–75)		0.43 (0.3–0.7)
Communication = 1	6	52 (38–66)	11 (2–28)			49 (46–52)		0.89 (0.8–1)

Acc, accuracy; Ext ASR, extinguishable auditory startle reflex; AUC, area under the ROC curve; NCS-r, Nociception Coma Scale-Revised; NLR, negative likelihood ratio; NPV, negative predictive value; PLR, positive likelihood ratio; PPV, positive predictive value; Pr, prevalence; Sen, sensitivity; Sp, specificity.

### NAR presence correlates with CNV presence

As mentioned above, all patients were also recorded with EEG while submitted to the active counting version of the auditory ‘local global’.^15,16,19^ One EMCS patient could not be recorded with EEG, and one EEG data set from a patient in the VS/UWS was rejected after automatic data quality assessment. EEG data were therefore available from 55 patients (23 patients in the VS/UWS, 28 patients in the MCS, and 4 patients in the EMCS; Supplementary Table 2).

**Figure 3:**
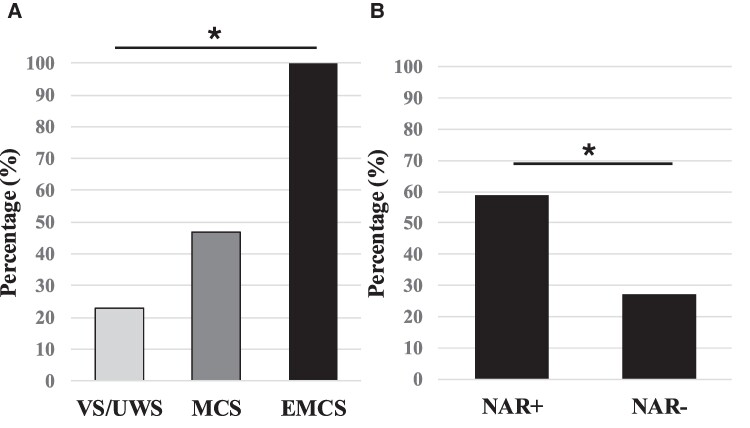
**Proportion of CNV according to clinical state (A) and NAR (B).** EMCS, emerging from minimally conscious state; MCS+, minimally conscious state plus; VS/UWS, vegetative state/unresponsive wakefulness syndrome. *P < 0.05 (*χ*^2^ in **A** and Fischer’s exact test in **B**).

As predicted, presence of a CNV was associated to NAR+ status: a significant CNV was found in 22 (40%) patients, including 13/23 NAR+ patients (57%) and in 9/32 NAR− patients (28%; Fisher’s exact test *P* = 0.05). CNV presence was also correlated with NTAR: 13/22 NTAR+ patients (59%) and in 9/33 NTAR− patients (27%; Fisher’s exact test *P* = 0.01) and to conscious status: 6/23 (23%) patients in a VS/UWS, 12/28 (43%) patients in a MCS, and 4/4 patients in an EMCS (4/4) (*χ*^2^  *P* = 0.02 and see Fig. 3).

*Post hoc* correlation tests between NAR and CNV restricted to each conscious status did not reach significance, probably due to the limited effectives. We then ran a logistic regression of CNV presence as function of NAR while declaring conscious status (VS or MCS− versus MCS+ or EMCS) as a covariate and found a significant effect of NAR+ (odds ratio = 3.1; *P* = 0.05).

In agreement with our previous reports,^15,19^ presence of a local effect was observed in 34 (61%) patients and did not discriminate VS/UWS versus MCS patients (11/23 versus 19/28; Fischer’s exact test *P* = 0.17), but this effect was found in each of the conscious EMCS patients (4/4). A local effect was more frequent for NAR+ than for NAR− patients [18/23 (78%) versus 16/32 (50%); Fisher’s exact test *P* = 0.05].

A global effect was present in 12 (22%) patients: in 5/23 (22%) patients in a VS/UWS; in 5/28 (18%) patients in a MCS; and in 2/4 (50%) conscious patients in an EMCS (*χ*^2^  *P* = 0.4). Presence of a global effect did not differ significantly between NAR+ and NAR− patients [NAR+ 6/23 (26%) versus NAR− 6/32 (19%); Fisher’s exact test *P* = 0.5; Supplementary Table 2]. Interestingly, the single patient in a VS/UWS who demonstrated pain anticipation (NAR+) had both a significant local and global ERP effects, suggesting a richer cognitive state than suggested by usual neurological examination and CRS-r scoring.

### NAR does not predict clinical outcome

At 6 months, 26/57 (46%) patients were dead: 9 were initially in a MCS (32%), 16 in a VS/UWS (66%), and 1 in a conscious EMCS (25%). As previously reported,^19,24,25^ initial status (MCS versus VS/UWS) strongly predicted consciousness recovery: while this major improvement was present for 50% (14) of MCS patients, it was observed in only 17% (4) of VS/UWS patients [AUC = 65% with 95% CI (51–78); Fisher’s exact test *P* = 0.02]. The same analysis ran on survivors did not reach significance, most probably because of a power issue explained by the high proportion of death in VS/UWS group (*P* = 0.37, Table 3). Presence of a NAR only showed a non-significant trend to predict consciousness recovery: 9/33 NAR− patients recovered consciousness, whereas 9/19 NAR+ patients did (47% versus 27%, Fisher’s exact test *P*-value = 0.2). Note that a similar trend was observed when comparing GOSE scores of NAR+ versus NAR− patients (NAR+ mean GOSE = 2.5 ± 1.3; NAR-mean GOSE = 2 ± 1.5 *P* = 0.15 in Wilcoxon–Mann–Whitney). This negative result may be also explained by a difference of power that should be checked in a larger cohort of patients.

**Table 3:** Pain anticipation predictive performance on consciousness recovery in survivors only

	AUC (95% CI)	Sen % (95% CI)	Sp % (95% CI)	PPV % (95% CI)	NPV. % (95% CI)	PLR (95% CI)	NLR (95% CI)
NAR+	59 (39–78)	50 (26–74)	78 (40–97)	82 (55–94)	44 (30–58)	2.2 (1–8)	0.6 (0.4–1)
Ext ASR	63 (42–81)	78 (52–94)	43 (10–82)	78 (63–88)	33 (7–70)	1.2 (1–2)	0.70 (0.2–2)
NCS-r (≥ 4)	76 (55–91)	83% (59–96)	57 (18–90)	83 (67–92)	57 (28–82)	1.9 (1–5)	0.3 (0.1–1)
MCS	67 (40–83)	78 (52–94)	44 (14–79)	74 (60–84)	50 (24–76)	1.4 (1–3)	0.5 (0.2–2)

## Discussion

In the present study, we designed and tested a new behavioural sign of MCS that can be easily tested at bedside: the NAR. We showed that presence of NAR discriminated EMCS and MCS from VS/UWS patients and therefore performed close to other CRS-r MCS discriminative items and close to the presence of extinguishable ASR that we validated in a previous study.^6^ This NAR was also congruent to presence of a NCS-r ≥ 4 (according to the threshold proposed by Chatelle *et al*.^5^). The single patient in a VS/UWS who demonstrated pain anticipation (i.e.: NAR+) had both a significant global ERP effect specific to MCS and EMCS, suggesting a richer cognitive state than suggested by usual neurological examination and CRS-r scoring. We know discuss several key aspects related to these results.

### A new behavioural sign of MCS:

This NAR+ appears as a new powerful bedside behavioural sign of MCS. This new sign fulfils several crucial criteria: first, it was present in all conscious patients (EMCS). Second, NAR+ was strongly associated to MCS, with a gradient following evidence strength of conscious processing: EMCS > MCS+ > MCS−, while it was present in only one patient in the VS/UWS (out of 24 patients in VS/UWS). Third, this NAR was found correlated to the CNV ERP component that indexes a preserved ability of stimulus anticipation. Interestingly, the single NAR + VS/UWS patient showed habituation of the auditory startle reflex, a new behavioural sign very specific of MCS,^6^ and he also showed a CNV as well as a global effect. In other terms, this patient was probably in a covert MCS.^3,20,26^ Taken together, our findings show that a NAR+ status is highly indicative of an overt or covert MCS state, and therefore that the presence of such a behaviour should question a behavioural diagnostic of VS/UWS. The simple hierarchical procedure we proposed could be implemented easily at bedside by trained caregivers. Patients with DoC are potentially exposed to numerous and various types of nociceptive stimuli during daily care or elicited by prolonged immobility.^23^ Because of limited or absent ability to communicate their potential subjective experience of pain, DoC patients are more vulnerable. Introducing a new tool, such as the NAR, can provide caregivers with an additional method to detect and assess potential pain experiences in these patients. Indeed, this cost-effective and simple procedure to explore pain anticipation at bedside can be implemented in a variety of healthcare settings, including resource-limited settings. Pain experience encompasses multiple aspects (sensory, cognitive, and emotional).^27^The sensory dimension of nociceptive stimulus is mainly processed by the lateral thalamic nucleus, the primary and secondary somato-sensory cortex, and the insula. The affective and cognitive dimension of pain is mainly handled by the medial thalamic nucleus, the prefrontal cortex, the anterior cingulate cortex, the insula, and the cingulate cortex .^28,29^ These last regions played a key role on subjective experience of pain.^30^ Interestingly neurophysiological and neuroimaging studies have measured a preserved cortical activity of brain regions within this large pain-related neuromatrix in some MCS patients,^31^ while in some VS/UWS patient, the activity was restraint to the primary somato-sensory cortex.^32^ However, an activation of both sensory and affective network was measured in until 30% of patients behaviourally labelled as VS/UWS.^33-35^ These last results imply to be all the more vigilant to recognize and manage pain in patient with DoC and therefore improving comfort, well-being, and dignity within the clinical care setting.^36^ The subjective nature of pain is a fundamental aspect of the pain experience that is defined by the International Association for the Study of Pain (IASP) as a subjective and first-person ‘unpleasant sensory and emotional experience’ in difference to nociception. Appreciating this experience from a third-person caregiver perspective is especially difficult in the absence of functional communication. Beyond the extraction of features from an afferent signal, subjective experience is a self-referenced construct that also integrates information related to past experiences, present context, and future implications. In this perspective, anticipatory projective behaviours that escape from the immediate present and can be maintained in time for inter-trial durations beyond one second may reveal the preservation of such pain experience. The reproducible behavioural manifestations of noxious anticipation (e.g. facial expressions, withdrawal, vocalization) observed in our paradigm—as in real-life care—support the preservation of expectation-induced modulation of pain in some DoC. This aspect largely underexploited must be considered in the care of these patients.

### Revealing the functionality of a cortical network enabling pain anticipation at bedside

The correlation between the NAR and the anterior midline CNV ERP components, recorded during the ‘local global’ auditory paradigm, strongly suggests that NAR+ patients have a preserved fronto-temporal network enabling stimulus expectation and anticipation.^15,37-42^ The present NAR behaviour and auditory CNV are two complementary measures: while CNV is affected by manipulations of attention and motivation,^43^ NAR, as tested in our procedure, requires motor capacities. Both signs probably correspond to the activation of a large network implicating in particular prefrontal and hippocampus cortices.^8,44-46^ NAR is probably a marker of the residual functionality of this fronto-temporal network but does not imply univocally a preserved conscious state.

### Enriching the repertoire of cortically mediated states

As for all other MCS items of the CRS-r, presence of a NAR does not necessarily implies that the patient is in a conscious state associated with a self-reportable subjective experience.^47^ It rather demonstrates the functionality of a cortical network required for this cognitively mediated behaviour. In this context, we previously proposed that MCS should rather be conceived as cortically mediated state (CMS). While it is not clear at all if any MCS patient is in a residual conscious state, presence of MCS items (including this new NAR+ behaviour) reveals without any doubt the contribution of specified cortical networks to overt behaviour.^1^ Therefore, NAR+ item enriches the current repertoire of behavioural findings that reveal residual functionality of cortical networks. We recently used PET deoxyglucose data to show that, indeed, presence of a given MCS item (i.e. auditory, visual, or motor) does not correlate with a common brain-scale fronto-parietal network typically associated to conscious processing, but rather with a specialized cortical network congruent with the corresponding MCS item: visual MCS patients had increased metabolism in occipital regions, motor MCS patients in somato-sensory regions. Only auditory MCS patients and patients with extinguishable auditory startle reflex did show increased metabolism beyond auditory cortices, to include a fronto-parietal network suggestive of a residual conscious state. In a future work, it should be interesting to explore the brain metabolism and brain structure (including diffusion tensor imaging brain MRI) correlates of NAR+ behaviour: would this behaviour only tap to a specialized expectation/anticipation cortico-subcortical network, or would it correlate to a fronto-parietal network?

### Neural requirements of NAR

From a neural perspective, trace conditioning requires the contribution of a global workspace architecture including fronto-parietal areas, anterior cingulate cortex (ACC) and medial temporal lobe structures.^9,44,48^ Patients with bilateral hippocampal damage typically fail to acquire trace conditioning while they can still succeed in acquiring delay conditioning.^9,48^ An elegant c-fos expression study performed in rodents confirmed that trace fear conditioning engaged anterior cingulate cortex activity^10^ whereas delay conditioning does not. This area is proposed to play a key role in conscious state by the Global Workspace Neuronal Theory (GNWT) of consciousness^47,49^ and by other theories.^50,51^ In healthy awake and conscious volunteers, it has been shown that delay conditioning occurred even when the relation between US and CS was not consciously represented, whereas trace conditioning required conscious access to the US–CS relation.^8,52^ Trace and delay conditioning paradigms have been tested at bedside in various clinical states and situations. For instance, trace conditioning has been reported under anaesthesia^53^ and sleep.^4,54,55^ Bekinschtein *et al*.^56^ used an eye-blink trace conditioning paradigm in a group of MCS and VS/UWS patients. In this last study, the amount of trace conditioning learning correlated with the degree of cortical atrophy and was a good indicator of recovery. Conditioning vanished in control volunteers under the effect of propofol anaesthesia. In the same perspective, a study using a decoding EEG index of conditioning in the auditory modality detected evidence of trace conditioning in 8 of the 29 post-anoxic comatose patients tested.^57^ Interestingly, all survivors completely recovered consciousness. More recently, Cortese *et al*.^58^ used galvanic skin response and heart rate variability to detect trace conditioning in a nociceptive paradigm: nine of the patients in a VS/UWS who were tested were interpreted as trace conditioning learners. Eight of the nine patients (89%) evolved into MCS. The function of consciousness in trace conditioning, remains debated among authors^11,13,59^ and this in particular because of the controversial relation of attention and consciousness.^60,61^ Attentional inhibition played a major role in disrupting awareness of the CS–US contingency and thus trace conditioning. Successful trace conditioning, widespread among a wider variety of species, may not be by itself sufficient but necessary to infer consciousness.

Taken together, this literature suggests that presence of trace conditioning in DoC patients should reveal the preservation of cortical network functionality including working memory areas closely related to GNW and to conscious processing.

### Why almost all NAR+ patients showed both a NDAR and a NTAR?

The fact that in our series, and in divergence with one of our predictions, almost all NAR+ patients showed both a NDAR and a NTAR remains to be explained. Given the rich animal and human cumulative and converging literature stating different neural circuits for trace and delay conditioning, the most probable explanation should point to our specific procedure and population of patients. While we respected the temporal gap (>1 s) that seems critical to elicit and test trace-conditioning, we speculate that our task tapped into high-level cognition even for the delay-conditioning like condition. Indeed—and as we explained in the *Materials and methods* section—we deliberately chose to use a very limited number of nociceptive stimuli (three trials only). This could indeed have required high-level cognition typically recruited during fast learning processes with a very few set of trials. This hypothesis could be further tested through different settings: (i) a replication with a larger set of training trials would predict a higher difference between NDAR and NTAR rates (but such a replication would introduce new ethical concerns by increasing the number of nociceptive and potentially painful stimuli in patients); (ii) a replication of this study with other brain activity and brain structure tools would therefore be relevant to precise the physiological and psychological meaning of a preserved NAR; (iii) finally a replication of this study in a set of patients located within episodic memory networks (in particular within hippocampus and prefrontal cortex regions) would be of interest.

### Toward a fractionation of conscious versus unconscious pain anticipation mechanisms

NAR probably combines multiple forms of anticipation that require cortical processing but that do not necessarily require conscious processing.^37^ In particular, we showed that a non-conscious CNV elicited by subliminal masked stimulus originated from temporal lobe regions^37^ only, whereas CNV elicited by consciously visible stimuli originated from both medial temporal lobe and midline prefrontal cortex regions. Inspired by this dissociation, we could aim at disentangling conscious from non-conscious pain anticipation. We tried here to fractionate unconscious (NDAR inspired by delay conditioning) from conscious (NTAR inspired by trace conditioning) but did not find many dissociations in this series of patients. As mentioned above, it is possible that the fast learning requirement we used in this basic bedside procedure required high-level cognition including working memory capacities. We may improve the procedure by decreasing the cognitive requirements for a NDAR in order to better dissociate conscious from non-conscious NAR. The conditioning protocol could also be improved by using a conditioned stimulus correlated with a neutral stimulus and using different sensory modalities for CS and US to disambiguate basic sensory cues versus higher-order learned anticipation.

A second limitation of our work stems from our definition of pain anticipation that was exclusively based on motor behaviour. The concomitant measurement of other somatic markers (e.g. modulation of heart rate, galvanic skin response, and pupillary diameter) or of brain activity (e.g. modulation of EEG) might have increased sensitivity of pain anticipation responses and might also have allowed to include patients lacking motor response to nociceptive stimuli. The integrity of somato-sensory, nociceptive, and motor functional networks was not assessed with neurophysiological recordings as somato-sensory or laser-evoked potentials or with metabolic imagery. However, to make this task easy to perform at the patient’s bedside, we opted for a behavioural measure that could be easily reproduced by any clinician. For the same reason, the stimulus applied to the patient was not standardized through the using laser stimuli, a thermode or calibrating the pressure force applied to the nail bed. This lack of standardization could also represent a potential bias.

A third limitation of our work relates to the number of patients (*N* = 57). Even if this group size is rather important as compared to previously related works,^6,62^ two findings suggests that we are lacking some statistical power. First, while we found a prognostic value of behavioural status (i.e. MCS/CMS versus VS/UWS) on clinical and consciousness outcomes, as well as a strong relation between NAR status (i.e. NAR+ versus NAR−), we did not find the expected triangular correlation between NAR status and clinical and consciousness outcomes. Second, even the MCS/VS status only showed a trend of significance on survivors only analysis, whereas this effect is usually observed on larger cohorts of patients.^19,24,25^ Taken together, these limitations suggest that a replication of the NAR procedure should be conducted on a larger cohort of patients with multiple measures for each patient. Indeed several studies showed the value of repeating behavioural measures such as CRS-r to increase diagnostic and prognostic performance.^63^ This replication should also include inter-rater reliability or test–retest reliability of the NAR measure, to confirm the clinical relevance of this new tool.

To conclude, the present results illustrate how new clinical signs may be inspired by cognitive and behavioural sciences and how they can be tested in DoC. NAR appears as a promising new sign to detect residual cortically mediated behaviour at bedside. However, our results do not allow us to demonstrate its strong relevance in daily clinical practice. Additional studies are required, exploring this new sign in a larger series of patients, and its testing procedure could be optimized.

## Supplementary Material

fcae311_Supplementary_Data

## Data Availability

The data that support the findings of this study are available from the corresponding author, (L.N.) upon reasonable request.
