# Supplementary material for: Pain anticipation is a new behavioural sign of minimally conscious state
Source: Brain Commun. 2024 Sep 16;6(5):fcae311. doi: 10.1093/braincomms/fcae311 (PMC11430917; doi:10.1093/braincomms/fcae311)
Supplement: fcae311_Supplementary_Data [file fcae311_supplementary_data.pdf]

Supplementary Materials

Num	State	Sex, Age	Etiology	Delay (d)	Neurotropic med	CRS-r A/V/M/O/C/A	NCS-r M/V/F	ASR	NAR	NTAR	Outcome
**1**	VS/UWS	M,71	anoxia	77	ZNS, VPA, LEV	6:1/0/2/1/0/2	3: 2/0/1	IN	no	no	MCS
**2**	VS/UWS	F,34	hypogly	38	LEV	4:1/0/1/1/0/1	1:1/0/0	IN	no	no	Death
**3**	VS/UWS	M,66	PRESS	40	-	7:1/1/2/1/0/2	3:2/0/1	IN	no	no	Death
**4**	VS/UWS	M,67	epilepsy	120	ZNS, VPA Benzo	8:2/1/2/1/0/2	4:2/0/2	EX	yes	no	Death
**5**	VS/UWS	F,50	anoxia	52	LEV	5:1/0/1/1/0/2	3:2/0/1	IN	no	no	Death
**6**	VS/UWS	F,40	stroke	30	LEV	3:0/0/2/0/0/1	2:2/0/0	abs	no	no	Conscious
**7**	VS/UWS	M,43	anoxia	73	Benzo	7:1/0/2/2/0/2	6:2/2/2	EX	no	no	VS/UWS
**8**	VS/UWS	F,41	hypogly	13	-	2:0/0/1/0/0/1	1:1/0/0	IN	no	no	Death
**9**	VS/UWS	M,78	anoxia	46	LEV	5:1/0/2/1/0/1	3:2/0/1	EX	no	no	Death
**10**	VS/UWS	M,49	anoxia	24	LCM,Benzo	4:1/0/1/1/0/1	1:1/0/0	IN	no	no	Death
**11**	VS/UWS	F,29	dys.enc	97	LCM	6:1/0/2/1/0/2	4:2/0/2	IN	no	no	Conscious
**12**	VS/UWS	F,61	anoxia	17	Morphine	6:1/1/2/1/0/1	2:1/0/1	IN	no	no	Death
**13**	VS/UWS	M,60	stroke	48	LEV, LCM,	6:1/1/2/1/0/1	4:2/0/2	EX	no	no	Death
**14**	VS/UWS	M,56	anoxia	62	SCP	6:1/0/2/1/0/2	1:1/0/0	IN	no	no	Conscious
**15**	VS/UWS	M,57	stroke	15	LEV	6:1/1/2/1/0/1	3:2/0/1	EX	no	no	Conscious
**16**	VS/UWS	M,21	anoxia	44	Morphine	7:1/1/2/1/0/2	2:2/0/0	EX	no	no	Death
**17**	VS/UWS	F,47	stroke	707	LEV	5:1/0/2/1/0/1	3:2/0/1	EX	no	no	VS/UWS
**18**	VS/UWS	M,53	stroke	26	VPA	7:1/1/2/1/0/2	3:2/0/1	EX	no	no	Death
**19**	VS/UWS	M,49	anoxia	22	LEV	6:0/1/2/1/0/2	2:2/0/0	IN	no	no	Death
**20**	VS/UWS	F,64	anoxia	23	LEV	3:0/0/1/1/0/1	1:1/0/0	abs	no	no	Death
**21**	VS/UWS	M,37	anoxia	372	LEV, Xenazine	6:1/0/2/1/0/2	4:2/0/2	EX	no	no	Death
**22**	VS/UWS	M,55	hypogly	56	SCP	6:1/0/2/1/0/2	3:2/0/1	IN	no	no	Death
**23**	VS/UWS	M,28	TBI	43	Morphine	5:1/0/1/1/0/2	1:1/0/0	IN	no	no	Death
**24**	VS/UWS	F,58	stroke	51	LEV	3:0/1/1/1/0/0	1:1/0/1	abs	no	no	VS/UWS
**25**	MCS-	F,41	anoxia	5852	LEV	10:1/0/5/2/0/2	3:2/0/1	abs	no	no	MCS
**26**	MCS-	F,31	anoxia	27	ZNS,LEV	10:2/3/2/1/0/2	6:3/1/2	EX	yes	yes	Conscious
**27**	MCS-	F,59	anoxia	13	-	14:2/3/5/2/0/2	4:2/0/3	EX	yes	yes	Conscious
**28**	MCS-	M,21	stroke	299	Morphine	9:2/3/2/1/0/1	5:2/0/3	IN	no	no	Death
**29**	MCS-	M,68	stroke	25	-	11:2/2/5/1/0/1	5:3/0/2	EX	yes	yes	Conscious
**30**	MCS-	F,53	infectious	68	LEV	14:2/3/5/2/0/2	8:3/2/3	EX	yes	yes	Conscious
**31**	MCS-	F,48	dys.enc	11	ESC	9:1/1/5/1/0/1	6:3/1/2	IN	no	no	Conscious
**32**	MCS-	M,63	stroke	28	-	10:2/3/2/1/0/2	4:2/0/2	EX	no	no	Conscious
**33**	MCS-	F,45	dys.enc	79	-	11:2/3/2/2/0/2	5:2/1/2	EX	yes	yes	Conscious
**34**	MCS-	F,61	anoxia	33	-	13:2/3/5/1/0/2	4:2/0/2	EX	no	no	MCS
**35**	MCS-	F,51	anoxia	12	LEV	11:2/3/3/1/0/2	6:3/1/2	EX	yes	no	Death
**36**	MCS-	M,21	TBI	124	LEV,Benzo	8:1/3/1/1/0/2	2:1/0/1	IN	no	no	Death
**37**	MCS-	F,25	TBI	544	LTG	12:2/3/4/1/0/2	5:3/0/2	EX	yes	yes	MCS
**38**	MCS-	M,34	TBI	245	SCP	9:1/1/4/1/0/2	5:3/0/2	EX	yes	yes	MCS
**39**	MCS-	F,36	infectious	76	-	9:2/2/2/1/0/2	4:2/0/2	EX	no	no	MCS
**40**	MCS-	M,33	TBI	120	GBP,Benzo	10:2/3/2/1/0/2	4:2/0/2	EX	no	no	Conscious
**41**	MCS-	M,55	anoxia	17	-	7:1/0/5/1/0/0	4:2/0/2	EX	no	no	Conscious
**42**	MCS+	M,56	infectious	56	Benzo	19:3/5/5/2/1/3	6:3/1/2	EX	yes	yes	Conscious
**43**	MCS+	M,61	infectious	51	-	11:3/3/2/1/0/2	5:2/1/2	EX	yes	yes	Conscious
**44**	MCS+	M,50	infectious	46	-	14:3/3/5/1/0/2	5:3/0/2	EX	no	no	Conscious
**45**	MCS+	F,54	anoxia	42	ZNG	12:3/3/3/1/0/2	5:3/0/2	IN	yes	yes	Death
**46**	MCS+	F,73	infectious	140	SCP, Morphine	14:3/3/4/1/0/3	8:3/3/2	EX	yes	yes	Death
**47**	MCS+	M,19	anoxia	156	Benzo	16:4/3/4/2/0/3	7:3/2/2	EX	yes	yes	Death
**48**	MCS+	M,59	TBI	219	Benzo,AML	13:3/4/2/2/0/2	4:2/0/2	EX	yes	yes	Death
**49**	MCS+	F,59	anoxia	23	Benzo	12:4/3/2/1/0/2	5:2/1/2	EX	yes	yes	Conscious
**50**	MCS+	M,52	anoxia	37	LEV,ZNG,Benzo	15:3/5/2/1/1/3	4:2/0/2	EX	yes	yes	Death
**51**	MCS+	F,71	infectious	97	LEV,LCM,Benzo	19:3/5/5/2/1/3	6:2/2/2	EX	yes	yes	Death
**52**	MCS+	M,56	TBI	478	AML	15:3/4/5/1/0/2	5:3/0/2	EX	yes	yes	Conscious
**53**	EMCS	M,18	TBI	124	LCM,Benzo	22:4/5/6/2/2/3	7:3/2/2	EX	yes	yes	Conscious
**54**	EMCS	M,34	TBI	109	AML	23:4/5/6/3/2/3	7:3/2/2	EX	yes	yes	Conscious
**55**	EMCS	M,28	epilepsy	106	LTG,VPA	19:4/5/5/1/1/3	7:3/2/2	IN	yes	yes	Conscious
**56**	EMCS	M,80	TBI	88	-	19:3/4/6/2/1/3	8:3/3/2	EX	yes	yes	Death
**57**	EMCS	M,63	stroke	64	CPZ	19:3/4/6/2/1/3	8:3/3/2	EX	yes	yes	Conscious

Supplementary Table 1. Patient characteristics Sex, Age, Aetiology, Delay of DoC in days, Sedative or neurotropic medications, CRS-r (A:Auditory, V: Visual, M: Motor,O: Oromotor, A: Arousal, C: Communication subscales) , NCS-r (M: Motor, V:Verbal, F:Facial subscales); auditory startle reflexe (IN : inextinguishable, EX ; extinguishable, abs : absent), NTAR ,NAR and 6 months outcome. Abbreviations: epilepsy = Epileptic encephalopathy, dys.enc.=Dysimmune PRESS= posterior reversible encephalopathy syndrome, TBI: Traumatic brain injury. Encephalitis, infectious= infectious encephalitis, Hypogly=Hypoglycemia, AML= Mantadix, Benzo= benzodiazepine, ESC= escitalopram, GBP= Gabapentine, LCM = Lacosamide, LEV=Keppra, LTG= Lamotrigine, SCP= Scopolamine, ZNS=Zonegran, VPA =Depakine

Num	State	NAR	NTAR	Local effect	Global effect	CNV	Outcome
**1**	VS/UWS	no	no	No	no	no	MCS
**2**	VS/UWS	no	no	no	no	no	Death
**3**	VS/UWS	no	no	yes	no	no	Death
**4**	VS/UWS	yes	no	yes	yes	no	Death
**5**	VS/UWS	no	no	no	no	no	Death
**6**	VS/UWS	no	no	no	yes	yes	Conscious
**7**	VS/UWS	no	no	no	no	no	VS/UWS
**8**	VS/UWS	no	no	-	-	-	Death
**9**	VS/UWS	no	no	yes	no	no	Death
**10**	VS/UWS	no	no	no	no	no	Death
**11**	VS/UWS	no	no	no	no	no	Conscious
**12**	VS/UWS	no	no	no	no	no	Death
**13**	VS/UWS	no	no	yes	no	yes	Death
**14**	VS/UWS	no	no	yes	no	no	Conscious
**15**	VS/UWS	no	no	no	no	no	Conscious
**16**	VS/UWS	no	no	no	no	yes	Death
**17**	VS/UWS	no	no	yes	yes	no	VS/UWS
**18**	VS/UWS	no	no	yes	yes	yes	Death
**19**	VS/UWS	no	no	yes	yes	no	Death
**20**	VS/UWS	no	no	yes	no	no	Death
**21**	VS/UWS	no	no	no	no	no	Death
**22**	VS/UWS	no	no	no	no	yes	Death
**23**	VS/UWS	no	no	yes	no	yes	Death
**24**	VS/UWS	no	no	yes	no	no	VS/UWS
**25**	MCS-	no	no	yes	no	no	MCS
**26**	MCS-	yes	yes	yes	yes	no	Conscious
**27**	MCS-	yes	yes	yes	no	yes	Conscious
**28**	MCS-	no	no	no	no	no	Death
**29**	MCS-	yes	yes	yes	no	yes	Conscious
**30**	MCS-	yes	yes	yes	no	no	Conscious
**31**	MCS-	no	no	yes	no	no	Conscious
**32**	MCS-	no	no	yes	no	yes	Conscious
**33**	MCS-	yes	yes	yes	no	yes	Conscious
**34**	MCS-	no	no	no	no	yes	MCS
**35**	MCS-	yes	no	yes	no	no	Death
**36**	MCS-	no	no	yes	yes	no	Death
**37**	MCS-	yes	yes	yes	no	no	MCS
**38**	MCS-	yes	yes	yes	yes	yes	MCS
**39**	MCS-	no	no	yes	no	no	MCS
**40**	MCS-	no	no	yes	yes	yes	Conscious
**41**	MCS-	no	no	no	no	no	Conscious
**42**	MCS+	yes	yes	no	no	no	Conscious
**43**	MCS+	yes	yes	no	no	no	Conscious
**44**	MCS+	no	no	no	no	no	Conscious
**45**	MCS+	yes	yes	yes	no	no	Death
**46**	MCS+	yes	yes	no	no	yes	Death
**47**	MCS+	yes	yes	yes	no	yes	Death
**48**	MCS+	yes	yes	no	no	yes	Death
**49**	MCS+	yes	yes	yes	no	no	Conscious
**50**	MCS+	yes	yes	yes	no	yes	Death
**51**	MCS+	yes	yes	yes	yes	no	Death
**52**	MCS+	yes	yes	no	no	yes	Conscious
**53**	EMCS	yes	yes	yes	yes	yes	Conscious
**54**	EMCS	yes	yes	yes	yes	yes	Conscious
**55**	EMCS	yes	yes	yes	no	yes	Conscious
**56**	EMCS	yes	yes	yes	no	yes	Death
**57**	EMCS	yes	yes	-	-	-	Conscious

Supplementary Table 2. NAR, ERP signatures of the 'local-global' auditory paradigm and 6 months outcome.
